# Bridging functional bionanomaterials and the clinic: strategic communication as a translational enabler

**DOI:** 10.1186/s12951-026-04425-y

**Published:** 2026-04-29

**Authors:** Dana-Mihaela Vrabie, Fang Yang, Aiguo Wu, Carlos Lodeiro, Pablo del Pino, Beatriz Pelaz, Georgeta Panisoara, Young Min Song, Marius Popescu, Stefan G. Stanciu, Gheorghe Militaru

**Affiliations:** 1 Doctoral School of Entrepreneurship, Business Engineering and Management, National University of Science and Technology Politehnica Bucharest, 313 Splaiul Independentei, 060042 Bucharest, Romania; 2 The Laboratory of Advanced Theranostic Materials and Technology, Ningbo Key Laboratory of Biomedical Imaging Probe Materials and Technology, Chinese Academy of Sciences (CAS) Key Laboratory of Magnetic Materials and Devices, Ningbo Institute of Materials Technology and Engineering, Chinese Academy of Sciences, Ningbo, 315201 China; 3 Zhejiang International Cooperation Base of Biomedical Materials Technology and Application, Zhejiang Engineering Research Center for Biomedical Materials, Ningbo Cixi Institute of Biomedical Engineering, Cixi, 315300 China; 4 BIOSCOPE Research Group, LAQV-REQUIMTE, Department of Chemistry, NOVA School of Science and Technology, Universidade NOVA de Lisboa, Caparica, 2829 − 516 Portugal; 5 PROTEOMASS Scientific Society, Costa da Caparica, 2825 − 466 Portugal; 6 Centro Singular de Investigación en Química Biolóxica e Materiais Moleculares (CiQUS), Universidade de Santiago de Compostela, Santiago de Compostela, 15705 Spain; 7 Faculty of Psychology and Educational Sciences, University of Bucharest, 90 Panduri Street, 050663 Bucharest, Romania; 8 School of Electrical Engineering, Korea Advanced Institute of Science and Technology (KAIST), 291 Daehak-ro, Yuseong-gu, Daejeon, 34141 Republic of Korea; 9 Bucharest Clinical Emergency Hospital, 8 Calea Floreasca, Bucharest, 014461 Romania; 10 Photon-X Spectrum Lab, CAMPUS Research Institute, National University of Science and Technology Politehnica Bucharest, 313 Splaiul Independentei, 060042 Bucharest, Romania; 11 Center for Microscopy-Microanalysis and Information Processing, National University of Science and Technology Politehnica Bucharest, 313 Splaiul Independentei, 060042 Bucharest, Romania; 12 Department of Management, Faculty of Entrepreneurship, Business Engineering and Management, National University of Science and Technology Politehnica Bucharest, 313 Splaiul Independentei, 060042 Bucharest, Romania

**Keywords:** Bionanomaterials, Nanomedicine translation, Clinical adoption, Strategic communication, Stakeholder engagement, Translational medicine, Nanobiotechnology, Innovation pathways

## Abstract

Functional bionanomaterials have enabled major advances in nanomedicine through their tunable physicochemical properties, multifunctionality, and ability to interact with biological systems in a controlled manner. Despite sustained progress in materials design and preclinical validation, the clinical translation of bionanomaterials remains limited, with only a small fraction achieving regulatory approval and routine clinical use. While technical performance, safety, manufacturing scalability, and regulatory complexity are widely recognized as key translational barriers, the role of strategic communication in shaping stakeholder understanding, trust, and adoption has received comparatively little attention. In this Perspective, we argue that strategic communication should be considered a core translational enabler for functional bionanomaterials rather than a downstream dissemination activity, as in current trends. We identify recurrent communication gaps between bionanomaterial developers and key stakeholders, including clinicians, patients, regulators, and investors, that contribute to misaligned expectations, heightened risk perception, and delayed clinical adoption. Drawing on examples from nanomedicine translation, we discuss how differences in language, decision-making frameworks, and perceived value across stakeholder groups, can impede progress even for technically mature platforms. Building on insights from translational medicine, behavioral science, and innovation studies, we propose a structured communication framework tailored to the specific challenges of functional bionanomaterials. This framework emphasizes early integration of communication planning, stakeholder-specific narrative framing, visual and digital tools to reduce cognitive complexity, and iterative feedback mechanisms. We further discuss practical approaches for evaluating communication impact using measurable indicators such as stakeholder awareness, trust, regulatory feedback, and clinical uptake. By reframing communication as a bidirectional and designable component of the translational pipeline, this Perspective aims to support more efficient pathways for bringing functional bionanomaterials from the laboratory to the clinic.

**Graphical abstract:**

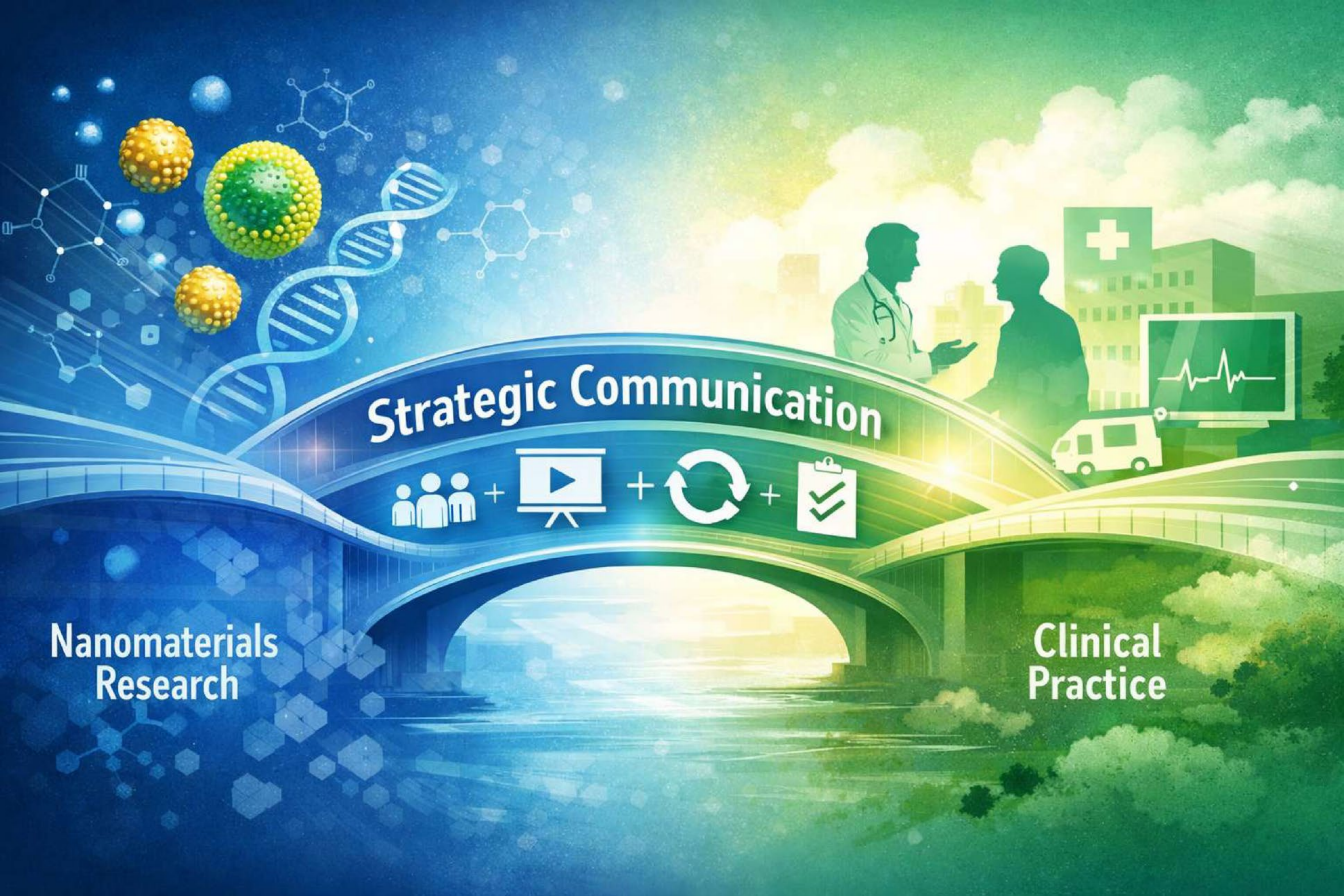

## Introduction

Functional bionanomaterials constitute a core class of technologies within the broader field of nanobiotechnology. They represent a central pillar of contemporary nanomedicine, enabling advanced therapeutic, diagnostic, and theranostic strategies through precisely engineered nanoscale structures capable of programmable interactions with biological systems. Advances in materials synthesis, surface functionalization, and nano–bio interface engineering have led to bionanomaterials with highly tunable properties, including targeted delivery, stimuli responsiveness, and multifunctionality [1–3].

**Fig. 1:**
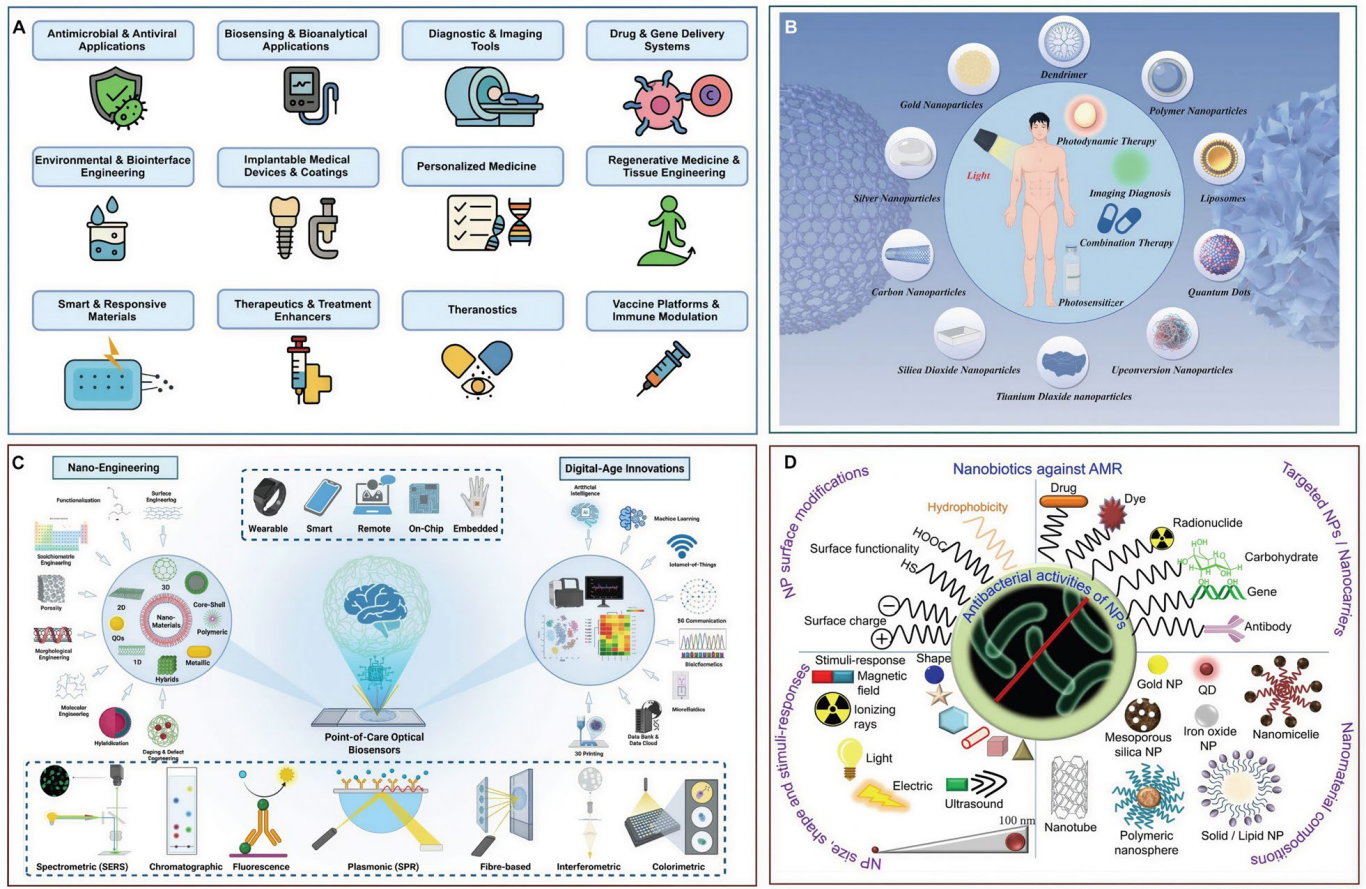
Applications and translational landscape of bionanomaterials. **A**) Overview of major application domains of bionanomaterials in nanomedicine and life sciences. **B**) Representative example of nanomaterial-enabled therapeutic and diagnostic strategies, highlighting the diversity of nanocarrier platforms and their integration into clinically relevant applications such as imaging, photodynamic therapy, and combination treatments. Reproduced from [36], available under CC-BY license. **C**) Graphical overview of the convergence between nano-engineering and digital-age technologies (e.g., artificial intelligence, wearable systems, and microfluidics) in the development of advanced biosensing platforms. Reproduced from [37] available under CC-BY license; **D**) Graphical representation of the design space of nanobiotics, illustrating how physicochemical properties, surface functionalization, and stimulus-responsive behaviors can be engineered to enhance antimicrobial activity and enable targeted therapeutic strategies. Reproduced from [38], available under CC-BY license

Today, bionanomaterials are positioned as key enablers of emerging clinical approaches across oncology, infectious diseases, and regenerative medicine. They serve as foundational building blocks for a wide range of biomedical applications, including therapy [4–7], diagnostics [8–10], theranostics [11–13], tissular regeneration [14–16], or biosensing [17–19], (Fig. 1). Engineered at the nanoscale to exhibit finely tailored bioactivity, responsiveness, and multifunctionality [6, 20–22] these materials hold considerable potential to transform medical practice. New applications in targeted drug delivery, implantable biosensors, and theranostic agents continue to emerge, addressing an increasingly broad spectrum of clinical needs [23].

Despite the significant progress outlined in the previous paragraph, which is primarily reflected at the level of scientific publications, the rate of successful clinical translation of bionanomaterials remains disproportionately low [24–28]. Frequently cited barriers include regulatory challenges [29–32] (e.g., difficulties in obtaining approvals for preclinical and clinical trials), scalability issues stemming from high production costs or poor reproducibility of a specific set of relevant physico-chemical parameters. Safety concerns related to the unexplored adverse effects of nanomaterials within the human body have also been widely debated and still represent a bottleneck [33–35]. All these barriers are well documented in the nanomedicine literature. However, the translation of bionanomaterials is not governed solely by technical and regulatory considerations. It is also shaped by how innovations are communicated, interpreted, and trusted by diverse stakeholders, including clinicians, patients, regulators, investors, and healthcare systems. Differences in professional language, risk perception, and decision-making priorities frequently result in misalignment between bionanomaterial developers and those responsible for evaluating or adopting these technologies. Therefore, even technically mature bionanomaterials may encounter resistance or delay in clinical integration.

Given the increasingly competitive landscape of innovation, the mere publication of scientific results in specialized journals, even in top-tier ones, has proven insufficient to propel developed solutions from the lab to the clinic. This is a key obstacle to consider, as the slow penetration of bionanomaterials into clinical biomedical applications, from a psychological point of view, faces obstacles rooted in fundamental/basic emotional and cognitive mechanisms that shape decision-making. In emerging technologies, uncertainty aversion often generates resistance, even when there is clear evidence of superior performance. Decision-makers rely on a cognitive and emotional evaluation of perceived benefits and risks, in which trust in the communicator can either play a crucial role or be completely disregarded.

**Fig. 2:**
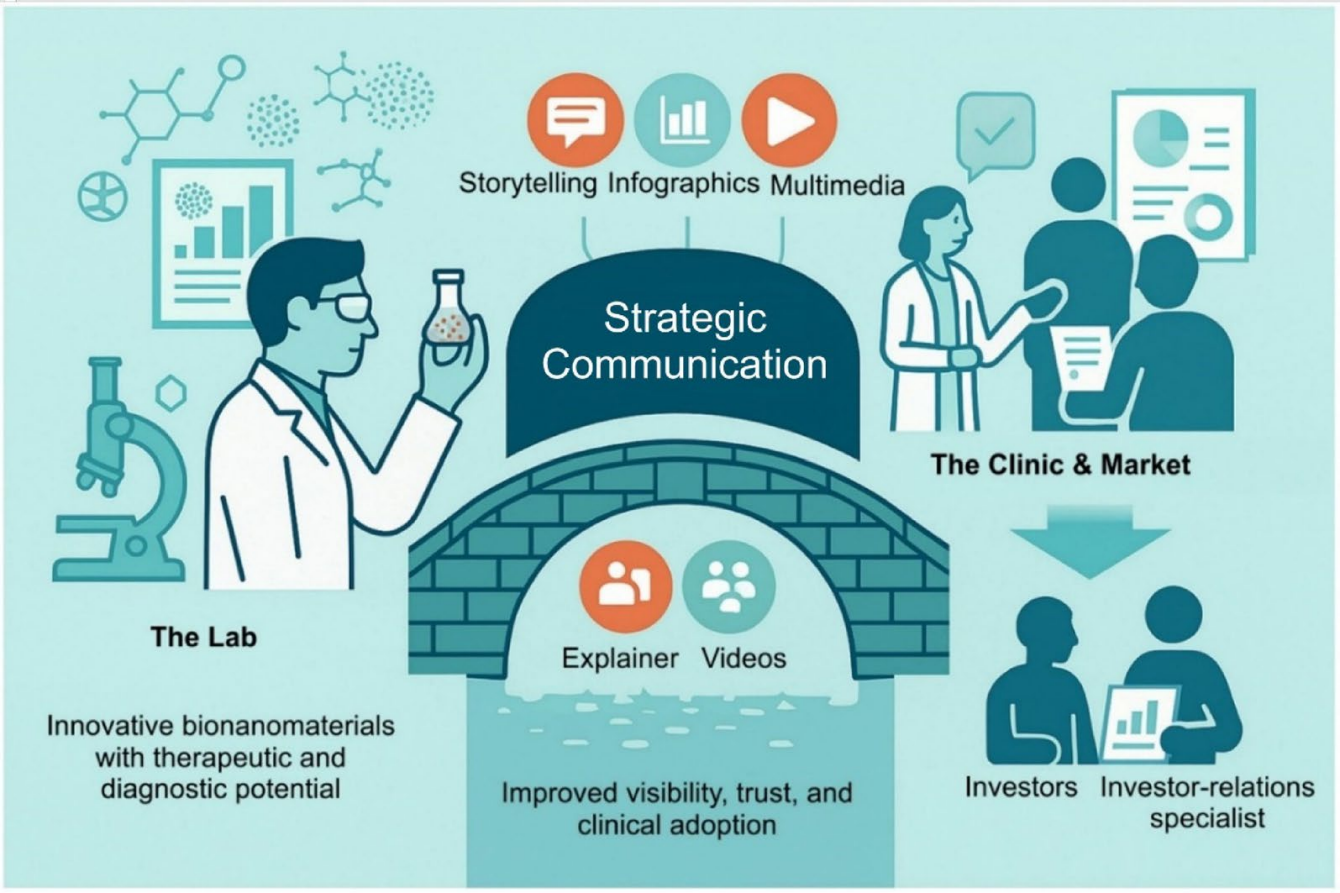
Strategic communication as a central pillar in the translational roadmap of bionanomaterials to the clinical practice

To overcome the barriers discussed above, there is a clear need for the development of strategic communication [39, 40] frameworks, workflows, and protocols capable of conveying the value, safety, and clinical relevance of bionanomaterials. In this context, throughout this Perspective, *strategic communication* refers to the intentional, structured, and stakeholder-oriented design of messages, channels, and feedback mechanisms that support informed decision-making, trust, and alignment across the translational pathway of functional bionanomaterials. Such approaches must be developed at multiple levels to address the needs of diverse stakeholder groups, including clinicians, patients, investors, and regulatory authorities (Fig. 2).

This Perspective therefore argues that strategic communication represents a critical yet underappreciated component of the clinical translation of functional bionanomaterials [41–43], one that should be considered a central pillar in the translational roadmap of bionanomaterials, Fig. 2. Rather than treating communication as a downstream dissemination activity, we propose that it should be embedded as a core translational enabler operating in parallel with scientific development and regulatory planning. After outlining key challenges in the current adoption landscape, we examine the role of strategic communication and describe specific strategies for targeted stakeholder engagement. Building on these considerations, we propose an integrated approach that draws on principles from strategic communication, behavioral science, and translational medicine to enhance the visibility, credibility, and clinical acceptance of emerging functional bionanomaterials. Finally, we discuss practical approaches for evaluating the impact of communication across the translational pipeline.

## Key challenges in the current adoption landscape

**Fig. 3:**
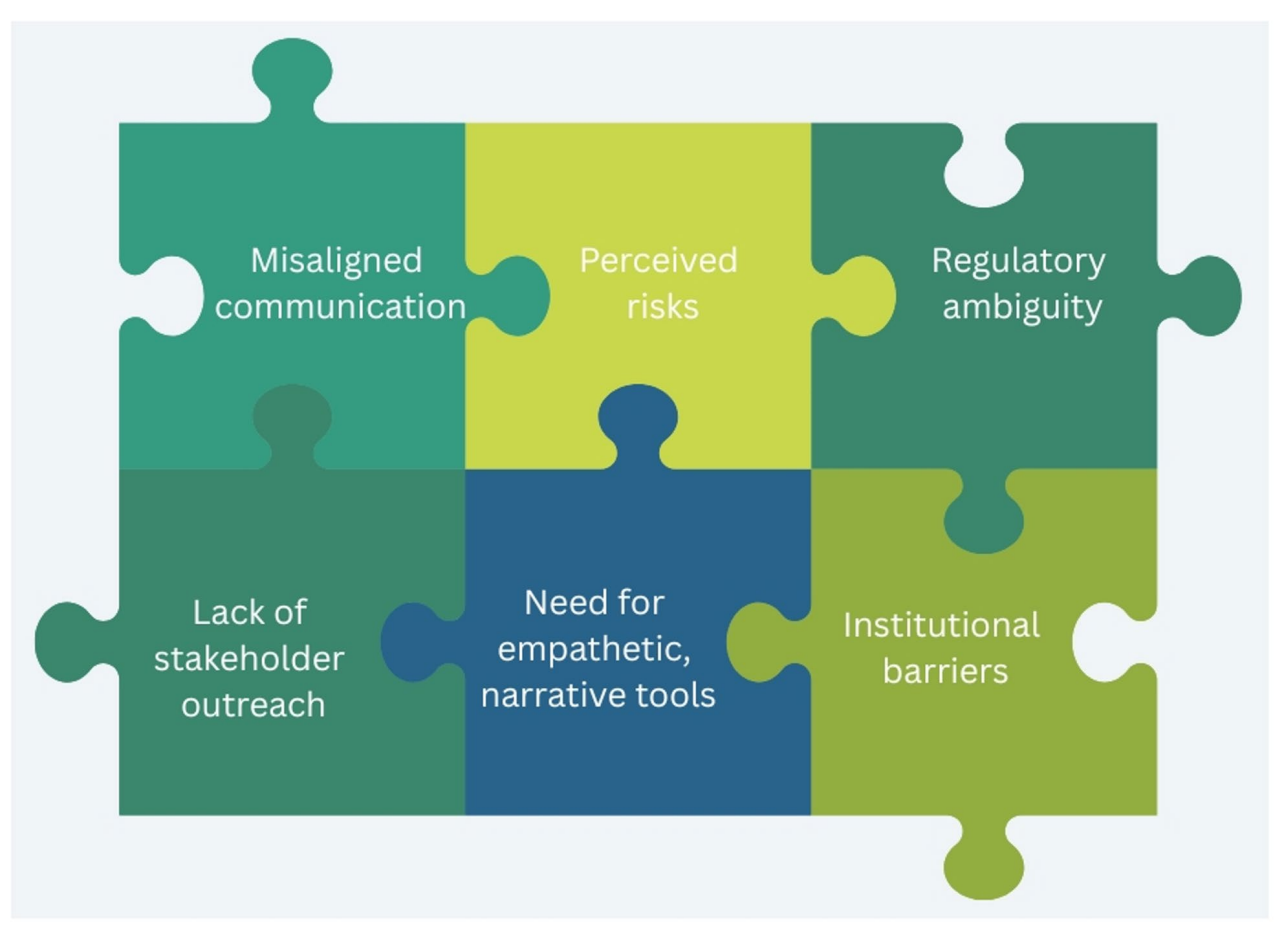
Key barriers to the translational adoption of functional bionanomaterials beyond technical readiness

The process of translating functional bionanomaterials from the status of laboratory innovation to the bedside, faces many additional obstacles beyond those of technical nature (e.g., performance, fabrication ease, costs, etc.). Below, we discuss some of these additional key challenges (Fig. 3), linked to communications.

### Misaligned communication between developers and translational stakeholders

One of the important hurdles in this journey is represented by communication barriers and inconsistencies [44–47] between scientific developers and key translational stakeholders. These represent a significant, yet frequently underestimated, issue. While scientists generate a vast number of high-impact publications and sophisticated prototypes, usually at low TRL level, there is a significant mismatch between what researchers consider to be “*clinically ready*” and what is understood, trusted, and accepted by end-users and regulatory bodies. A key step in addressing this situation has been made by the DELIVER framework proposed in 2024 by Joyce et. al. [48], comprising the core principles to be completed during preclinical development to promote clinical investigation of nanomedicines, but adoption of this framework at worldwide level is expected to pose significant challenges in the years to come, due to a wide palette of existing barriers [49, 50].

A key challenge stems from the notable differences in communication norms across the various disciplines involved in this discussion. For instance, clinicians and biomedical decision-makers prioritize aspects such as clinical utility, risk-benefit profiles, cost-effectiveness, and alignment with existing care protocols. When introduced to a new solution, such as a new functionalized bionanomaterial tailored for a given application, they will look in the pitch of the developers for keywords on such aspects. In contrast, scientific communications, usually written by the researchers who developed the new bionanomaterial or application, tend to emphasize novelty, mechanistic details, and the complexities of nanomaterial engineering, using highly specialized, domain-specific language and jargon. This style of communication aligns with what is typically valued in academic contexts like manuscript and proposal writing and reviewing. This disconnect between the delivered message, the differences between the two kinds of communicators, and the gaps in expectations of the different audiences often leads to outcomes that are overlooked or misunderstood due to the absence of a shared clinical versus scientific framework of communication. For example, the mRNA vaccine technology is the culmination of long-term scientific effort rather than a rapid overnight innovation [51], which many patients feared. However, during the mRNA vaccination campaigns within the COVID-19 pandemic bringing up such aspects were overlooked by the authorities, who mainly focused on disseminating technology aspects that were hard to grasp by the population. Whereas researchers emphasize scientific rigor of the methodology and conceptual innovation, the target audience responds more effectively to narratives that imply trust and easy understanding.

### Perceived risks, complexity, and public trust challenges

An additional challenge lies in the perceived risks associated with nanomedicine [47, 52, 53]. The inherent high complexity of nanomaterials, whose development often involves multifunctional platforms with hybrid organic-inorganic architectures can contribute to concerns about their unpredictability and safety. This perception is shared not only by clinicians and regulators but also patients [54, 55], who may relate complexity with risk. This issue is exacerbated by the lack of standardized communication strategies targeting safety, long-term biocompatibility, and reproducibility. A particularly relevant example is the social pharmacology study of Schultz et al. [56], which tested the hypothesis that individuals with a high level of familiarity with the healthcare system and greater trust in institutions would be inclined to view new drugs as more effective than old ones. However, only 25% of respondents agreed that new drugs are always more effective. The authors concluded that *“while drugs and vaccines with proven efficacy allow for sound health policy*,* pharmaceutical regulation failures and the overwhelming role of the pharmaceutical industry in drug development have created legitimate suspicion in the general public”*, which well reflects some of the reasons behind the sceptical public perception on novel nanomedicine tools and biomedical nanomaterials [57, 58].

Interestingly, even for specialized population such aspects on the perception of nanomedicine exist. For example, Nassani et al. [59], have assessed the perception, knowledge, and attitude of medical residents towards nanomedicine. Although most respondents expressed a positive attitude, the majority reported insufficient access to reliable information, which directly affects their perception of risk. The perception of potential risks is also amplified by historical controversies around nanotoxicity, a topic that is still under intense debate, still holding many opened questions and underexplored aspects, despite considerable advances in nanomaterials characterization and safety assessment. Moreover, cultural and religious beliefs, trust in science, and media representation play a crucial role in shaping the public’s perception of nanotechnology [60].

### Insufficient stakeholder-specific outreach and fragmented public engagement

Another important challenge is the lack of stakeholder-specific outreach. For example, both startups (e.g. spin-offs of academic origin) and academic groups that are involved in the development of functional bionanomaterials will most often communicate through preprints, peer-reviewed publications or technical conferences. Some of these dissemination channels are largely inaccessible, and most are poorly monitored, by non-academic stakeholders. In the absence of tailored messaging, appropriate communication channels and strategic framing, crucial audiences such as investors (who assess value and scalability), patients (who require trust and clarity), and healthcare administrators (who prioritize integration and cost) remain disengaged from most developments in the field, and have a poor understanding of the current state of the art [61]. Citizen science projects have been identified as useful resources to aid in achieving a greater degree of public understanding of science [62], but they cannot be regarded as a universal solution to this problem, which needs to be remedied from “upstream”. In this context, it is also important to recall some of the conclusions of the introduction to the 2013 Sackler Colloquium on “The Science of Science Communication,” where B. Fischhoff and D.A. Scheufele state [63]: *“Effective science–public communication depends*,* in part*,* on foundations laid years earlier. The more that laypeople have absorbed in science classes and informal science education*,* the better chance they have of grasping the science relevant to the decisions that they face. The more effectively scientists have built bridges with the rest of society*,* the better chance they have of getting a hearing for their work.”.* Against this backdrop, it is essential to emphasize the value of introducing children [64] to core concepts in nanotechnology from an early age, when they can start to understand key ideas presented in familiar contexts, such as how nanomaterials incorporated into everyday objects can make a difference.

### Regulatory ambiguity and the need for proactive communication with agencies

The regulatory landscape for bionanomaterials remains fragmented and can further exacerbate the challenges outlined in the previous [65, 66]. Many nanotechnology-based biomedical products, including ones relying on functional nanomaterials, exhibit hybrid characteristics, combining elements of drugs, medical devices, and biologics, as outlined in past landmark works [67–69]. A historical example that illustrates this complexity stands in superparamagnetic iron oxide nanoparticle (SPION) formulations developed as MRI contrast agents. These exhibit both pharmacological and device-like imaging functions [70–72], highlighting the difficulty of assigning such technologies to a single regulatory category. Due to such nuances and complexities, determining the appropriate regulatory classification and evaluation pathway may not always be straightforward. Moreover, regulatory approaches may vary across geographical regions, further complicating the classification and development of such technologies, as discussed by Rodríguez-Gómez et. al [29, 73].

This classification ambiguity can complicate development strategies, as different regulatory categories are associated with distinct approval processes, data requirements, and evaluation criteria. Consequently, developers may face uncertainty regarding the most appropriate regulatory framework for their technology.

For this reason, early and proactive engagement with regulatory agencies is essential. Structured dialogue between developers and regulators can help clarify the regulatory classification, align expectations regarding safety and efficacy assessments, and reduce the risk of delays during later stages of development.

Without such communication, even highly promising bionanomaterial platforms may experience significant delays in progressing along the lab-to-clinic translation pathway due to regulatory uncertainty rather than scientific limitations.

### The need for empathic, narrative-driven, and bidirectional communication tools

To address the communication challenges outlined above requires not only novel dissemination strategies, but also the development of new translational concepts that operate at multiple levels: the cognitive (simplifying complex or novel concepts), the social (aligning with group values and shared understanding), and the affective (creating emotional resonance). To achieve this, highly complex and multidimensional scientific innovations need to be transformed into appealing narratives, visuals, and messages that resonate with each stakeholder’s priorities, concerns, and level of understanding. In this context, empathic design plays a critical role. By incorporating an understanding of users’ emotional, cultural, and cognitive perspectives, it ensures that communication strategies are not only informative but also genuinely relatable and human-centered. Empathic design principles, long established in product development and innovation research [74, 75], can thus be effectively adapted to the translational ecosystem of bionanomaterials, guiding the creation of messages and interfaces that foster trust, relevance, and acceptance across diverse audiences. According to the Narrative Transportation Theory [76], people are more likely to change attitudes or understand complex information when they are emotionally and cognitively “transported” into a story. Such approaches would perfectly complement important efforts already made to popularize nanomaterials and nanotechnology [64, 77]; for example, the Knowledge Base Materials [78], a web-based information platform on nanomaterials (www.nanoobjects.info) aims to be constantly expanded. However, without downsizing their importance, such tools would greatly benefit from being designed as bidirectional and featuring interactive feedback, allowing stakeholders not only to receive information but also to contribute insights. To this end, the now-trending Large Language Models (LLMs) [79, 80] can represent a promising solution to endow such platforms with adaptive dialogue capabilities, tuned to each user’s background and educational level. Such approaches lie in the near horizons, with relevant efforts starting to emerge. For example, the recently introduced NANOGPT platform [81], is a specialized LLM-RAG system for nanotechnology research that integrates multi-source scholarly retrieval to deliver faster, more accurate, and more comprehensive literature reviews than standard publicly available LLMs.

### Internal institutional barriers to effective communication

An additional and often overlooked barrier lies within research institutions themselves. In many centers the responsibility for engagement and communication is left almost entirely to individual researchers, professionals who, while experts in their scientific domains, are often not trained or resourced for sustained stakeholder interaction. This structural gap significantly limits the translational impact of research outputs. Establishing dedicated, well-funded communication units within research institutions could provide the necessary expertise to support effective dissemination, stakeholder dialogue, and public engagement. Importantly, these structures require sustained financial and policy support. The burden of communication should not rest solely on researchers but should be recognized as a shared responsibility of funding bodies, governmental agencies, philanthropic organizations, and private investors alike, who have a vested interest in maximizing the efficiency, visibility, and societal return of the research they support. Furthermore, both public and private research institutions could greatly benefit from adopting proven communication frameworks from the pharmaceutical and biotechnology sectors, which have extensive experience in building trust, segmenting audiences, and conveying complex innovations to diverse societal groups [82, 83].

## The role of strategic communication

The effective translation of functional bionanomaterials increasingly requires not only technological advancement but also clear, credible, and strategically crafted communication. Strategic communication, when applied in a scientific and stakeholder-oriented manner, can help articulate clinical relevance, enhance trust, and reduce the perceptual and informational barriers that often hinder adoption. We have identified four complementary pillars: (i) strategic positioning, (ii) branding and trust building, (iii) insights from adjacent technological domains, and (iv) anticipatory behavioural communication strategies, that we regard as valuable for providing a structured foundation for conveying the value of emerging nanotechnologies to clinicians, patients, regulators, and investors. Together, these elements support a more coherent and impactful communication pathway throughout the translational process, as discussed in the next.

### Strategic positioning, value proposition, narrative framing and public familiarity

Strategic communication [39, 40] (drawing selectively also from marketing communication literature [42, 84, 85]) is traditionally associated with commercial sectors and is a field defined by a rich history. It is increasingly acknowledged as a highly effective means of shaping perceptions, mitigating uncertainty, and accelerating adoption within complex innovation ecosystems. In the context of functional bionanomaterials, strategic communication must address many additional aspects beyond the mere promotion of a particular technical solution. It requires carefully crafting appealing narratives, visuals, and engagement strategies that collectively translate the scientific merit of a given bionanomaterial or application into perceived clinical value. Importantly, this needs to be done for diverse audiences, clinicians, patients, regulators, and investors alike, each category requiring personalized media, tools, and strategies. For example, companies developing wearable medical devices achieved early adoption not by emphasizing the sophistication of their sensor technologies, but by using patient-centred storytelling that highlighted real-life benefits, ease of integration into clinical workflows, and partnerships with healthcare professionals [86–88]. This approach made complex innovations tangible and trustworthy, demonstrating how strategic narratives can transform technological novelty into relatable value propositions, a lesson highly relevant to the translational journey of bionanomaterials.

At its core, strategic communication is about the positioning of a given product within the landscape of the market it addresses. It begins with defining a convincing value proposition that aligns relevant features of a bionanomaterial, such as efficiency, precision targeting, controlled release, or biodegradability, with unmet clinical needs. Matching the innovative properties of bionanomaterials to specific clinical gaps requires consistent, credible messaging that emphasizes real-world relevance in addition to scientific novelty. A clear illustration of this principle can be found in the case of mRNA vaccines, which gained global traction once communication strategies shifted from emphasizing their molecular novelty to focusing on rapid production, scalability, and their crucial role in pandemic response [89, 90]. This reframing made a complex scientific platform comprehensible and compelling to both clinicians and the public. Similarly, novel bionanomaterials proposed as solutions to specific clinical pain points, such as reducing off-target effects in chemotherapy or improving biomarker detection thresholds, are far more likely to gain attention from stakeholders than those presented solely through their technical attributes (e.g., higher surface-to-volume ratios or dual-therapeutic pathways), since such advantages are more easily grasped by broader audiences.

### Branding and trust building

An aspect that is often overlooked by new companies, e.g., spin-offs with academic involvement, is that branding plays a subtle but important role in the frame of marketing communication [91, 92]. As in typical mass-consumption fields such as food, electronics, or clothing, in the scientific and medical domains as well, a recognizable and trusted identity is highly important for differentiating one technology from another [93, 94]. A key to making a biotech venture successful lies in the adoption of branding strategies that blend scientific credibility with emotional resonance. With respect to the latter, the branding of the product needs to project confidence, safety, reliability, affordability, ease of use and advantages compared to other products, among others. A telling example is the case of Doxil (liposomal doxorubicin), one of the first FDA-approved nanomedicines, Box 1. Despite its clear pharmacological advantages, Doxil initially experienced slow clinical uptake partly because early communication efforts failed to differentiate it convincingly from standard doxorubicin. Only when later campaigns emphasized its reduced cardiotoxicity and improved patient outcomes perceptions started to shift, highlighting how effective branding and framing can directly influence acceptance and market penetration [95]. For academic groups and early-stage spin-offs working on bionanomaterials, this can be achieved through a consistent visual identity, compelling multimedia materials that present the advantages of their innovations compared to the state of the art, and a constant media presence across diverse channels. Importantly, recognition and trust across time and platforms must remain a continuous focus of dissemination efforts.

**Table:** 

Box 1: Liposomal doxorubicin (Doxil®^®^) — delayed clinical uptake despite technical success Liposomal doxorubicin (Doxil®^®^) represents one of the earliest clinically approved nanomedicines, offering reduced cardiotoxicity and improved pharmacokinetics compared to free doxorubicin [95, 96]. Despite strong preclinical and clinical evidence, early clinical adoption was slower than expected. Retrospective analyses suggest that this delay was not solely due to technical or regulatory issues, but also to insufficient communication of its differentiated clinical value to oncologists. Initial messaging emphasized formulation novelty rather than concrete patient-relevant benefits such as toxicity reduction and improved tolerability. Subsequent reframing toward outcome-oriented narratives contributed to broader acceptance, illustrating how communication strategy can modulate the translational trajectory even after regulatory approval.	

### Lessons from adjacent technologies

Case studies from adjacent fields provide valuable lessons and have high potential to improve the adoption of a given product. For example, in the field of wearable medical devices, many companies have leveraged storytelling (e.g., user-centered design narratives) and collaborations with clinicians to drive early adoption [86–88]. Similarly, mRNA technology, long viewed as a feasible and elegant but clinically uncertain innovation, saw rapid adoption during the COVID-19 pandemic, partly due to strong messaging around its benefits, safety, and scalability [97–99], Box 2. The mRNA vaccine experience clearly illustrates how effective communication, when aligned with societal urgency and stakeholder trust, can reshape both public and investor perceptions almost overnight, transforming a once-niche technology into a global healthcare cornerstone [100]. These successes were possible by augmenting technical readiness aspects with well-orchestrated communication campaigns that engaged a broad spectrum of stakeholders, which was highly useful in accelerating their adoption.

A further relevant example can be found in the rapid clinical acceptance of AI-assisted radiology tools [101, 102]. Although early iterations were often met with skepticism [103] due to concerns about accuracy, workflow disruption, and “black-box” decision-making, adoption increased substantially once developers reframed their communication strategies. Instead of promoting the algorithms’ technical sophistication, successful companies emphasized co-pilot narratives, positioning AI as a supportive assistant to clinicians rather than a replacement [104]. This shift was complemented by transparent communication on validation datasets, error cases, and clinician oversight requirements, which collectively reduced perceived risk and strengthened professional trust. The trajectory of AI-based radiology software demonstrates how reframing disruptive technologies through collaborative, and clinician-centered messaging, can significantly accelerate acceptance, an approach directly applicable to the translational journey of bionanomaterials.

**Table:** 

Box 2: Lipid nanoparticle–based mRNA nanomedicines — rapid translation under aligned communication Lipid nanoparticle (LNP)–enabled mRNA vaccines represent a landmark success in nanobiotechnology translation [105, 106]. While decades of prior work established the technical feasibility of LNP-mediated delivery, widespread clinical acceptance accelerated only when communication strategies shifted toward clear articulation of safety, manufacturability, and clinical impact. During the COVID-19 pandemic, coordinated messaging by developers, regulators, and clinicians emphasized familiar concepts such as rapid scalability and transient biological action, reducing perceived novelty-related risk. This case illustrates how aligned, stakeholder-specific, communication can dramatically accelerate translation when technical readiness is already in place [90, 107, 108].	

### Anticipatory and behavioral communication strategies

In the case of bionanomaterials, strategic communication must also pre-emptively tackle resistance. This can be done by proactively addressing safety concerns, explaining mechanisms of action in accessible language, and providing comparative data with existing standards of care. By addressing such aspects, meant to settle the concerns of the stakeholders, even before these are formulated, a favourable perception of a given bionanomaterial or application can be shaped in an early stage of the translational pipeline. This anticipatory communication builds what behavioural economists call “cognitive fluency”, making complex concepts easier to understand and accept. Cognitive fluency is acknowledged as an important feature to address in marketing campaigns worldwide [109–111].

Ultimately, communication should not be viewed as one of the final steps on the journey of a given bionanomaterial from lab to hospital, but as a parallel process that evolves with the science and is continuously tuned, in synchronicity with the technical advances. This can be done by embedding communication planning into the research and development workflows. This way, by the time a bionanomaterial will meet the technological readiness level required for clinical trials, it will also be well-positioned to be understood, accepted, and supported by those who will ultimately determine its fate in the clinic. To this end, we refer again to the mRNA vaccines developed during the COVID-19 pandemic crisis. While post-development communication strategies were effective in the adoption of this new technology [89, 90], the same process was hindered by the lack of appropriate communication activities during the development of these vaccines since the 1990s when the first mRNA vaccines were first reported at preclinical level.

## Targeted strategies for stakeholder engagement

Strategic communication can influence translational outcomes through several mechanisms. First, transparent and consistent messaging can strengthen trust [112–115] among key stakeholders, including clinicians, regulators, investors, and patient communities. This dynamic was highlighted in the recent work of Kerr et al. [116], examining how trust-building communication practices shape stakeholder engagement in biomedical innovation. The authors examined how reframing traditionally persuasive messages according to “evidence communication” principles, such as presenting balanced risks and benefits, disclosing uncertainties and evidence quality, and prebunking common misperceptions, can improve science communication. Second, communication strategies influence how risks and benefits associated with emerging technologies are perceived [117, 118], which can affect both regulatory evaluation and public acceptance [119, 120]. For instance, an empirical study presented by Kamarulzaman et al. [120], has shown that communication approaches emphasizing transparent and balanced presentation of risks, benefits, and uncertainties can significantly enhance trust and acceptance of emerging nanotechnologies. Third, structured engagement with stakeholders [121] can facilitate alignment with regulatory expectations and clinical needs, thereby reducing uncertainty during development pathways, as highlighted in recent analyses [122] emphasizing the role of early multi-stakeholder dialogue in translational nanomedicine. Finally, clear articulation of the clinical value proposition [123, 124] of new technologies can support their integration into clinical practice by improving awareness and understanding among healthcare professionals [125, 126]. Through these mechanisms, communication acts as a complementary driver of technology adoption alongside technical performance and regulatory approval [124]. The mechanisms through which communication influences translational outcomes are summarized in Table 1.

Importantly, communication should not be viewed as an isolated driver of technology translation. Rather, it operates as a cross-cutting enabler that interacts with technical maturity, regulatory facilitation, and market dynamics by shaping how stakeholders perceive technological readiness, risks, and value propositions. Through these interactions, communication strategies can influence how emerging technologies progress through the translational pathway. In this sense, communication acts as a mediating layer between technological development and stakeholder decision-making (Table 1), influencing how technical evidence is interpreted, evaluated, and ultimately adopted within clinical and commercial ecosystems.

**Table 1:** Mechanisms through which strategic communication influences translational outcomes in bionanomaterials

Communication mechanism	Target stakeholders	Translational effect
Transparent messaging about risks and benefits	Public, patients, regulators	Builds trust and improves technology acceptance
Framing of technological value and clinical benefit	Clinicians, healthcare systems	Facilitates clinical uptake and integration into practice
Early engagement with regulatory agencies	Regulators	Aligns development with regulatory expectations and reduces uncertainty
Clear articulation of the value proposition	Investors, industry partners	Supports funding decisions and commercialization pathways
Accessible explanations and visual communication	Broader audiences	Reduces misinterpretation and improves understanding of emerging technologies

While strategic communication can facilitate stakeholder engagement and support the translational trajectory of emerging technologies, it also carries potential risks if not implemented responsibly. In particular, communication practices that emphasize promotional narratives over evidence-based messaging may contribute to unrealistic expectations regarding technological readiness [127], potentially reinforcing “hype cycles” in emerging technology domains [128]. Previous research reported by Van Lente et al. has shown that collective expectations and communication practices play a central role in shaping the rise and evolution of such technological hype cycles [129]. Such dynamics may create ethical tensions between marketing logic and scientific rigor, particularly when early-stage findings are communicated beyond specialist audiences. Maintaining transparency, accurately representing technological maturity, and prioritizing evidence-based communication are therefore essential for preserving credibility and trust in the development of novel bionanomaterials [130], particularly when communicating emerging technologies at the interface of scientific innovation and market expectations. To this end, the Review-paper of Singh et al. [131] emphasizes that transparent and realistic communication of technological capabilities and maturity is crucial for building credibility and facilitating the successful translation of nanomaterial-based technologies from laboratory research to practical applications.

**Scheme 1:**
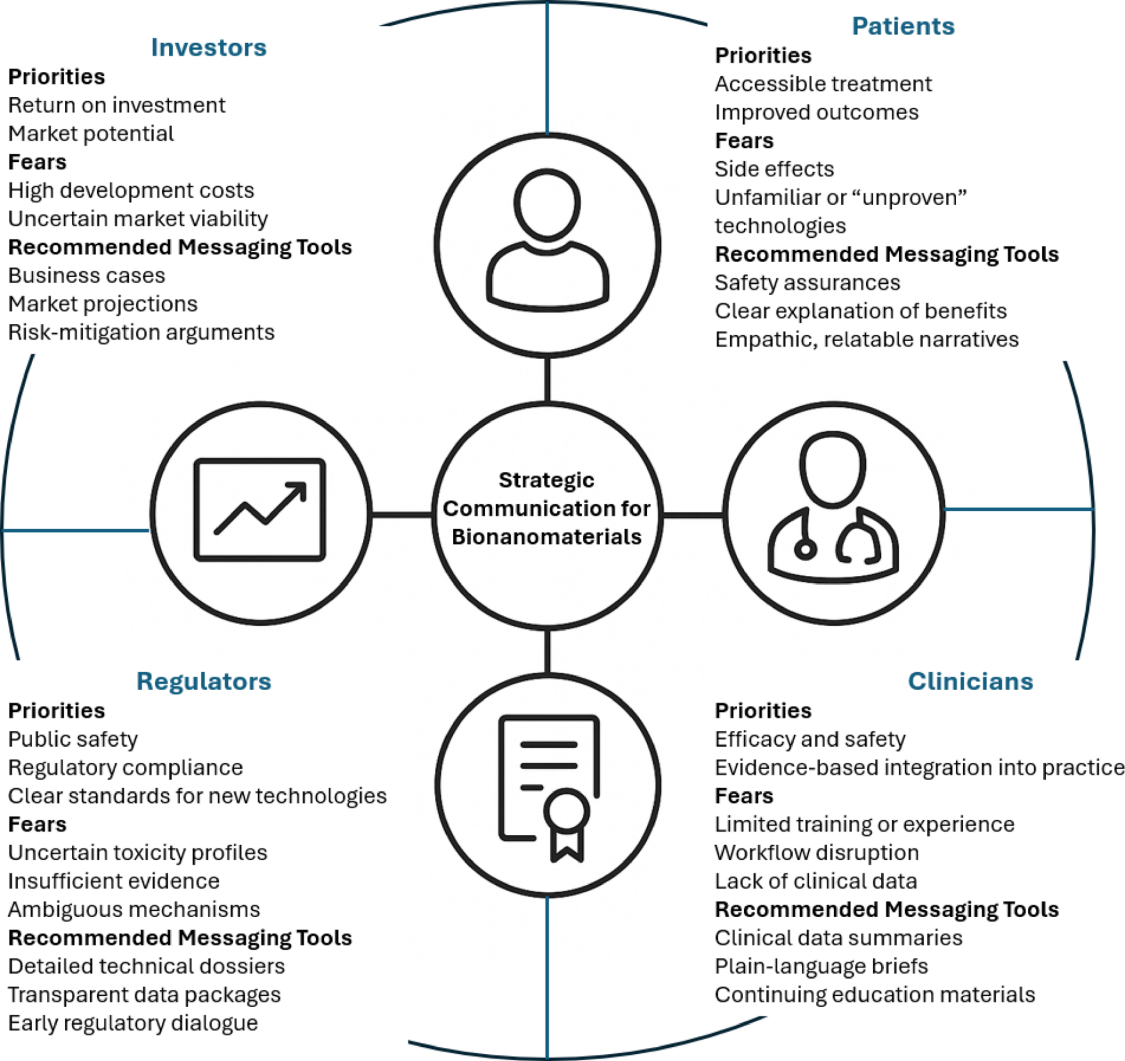
Radial stakeholder framework illustrating the key audiences involved in the translational adoption of bionanomaterials

As outlined in the next sub-sections, the translation of bionanomaterials from lab to clinic involves a diverse network of stakeholders, each playing a critical role at different stages of the process. Researchers and academic institutions drive discovery and early validation, often supported by public and private funding agencies. Regulatory bodies ensure safety and compliance, while industry partners handle scale-up, manufacturing, and commercialization. Clinicians and healthcare providers contribute practical insights and facilitate clinical trials, with patients and advocacy groups offering perspectives on acceptability and unmet needs. Payers and health technology assessment bodies influence adoption through reimbursement decisions, and ethics committees safeguard patient rights. Throughout, science communicators and marketing professionals need to play a key role, helping build understanding and trust. This requires the advent of tailored communication strategies that reflect the specifics of each stakeholder (Scheme 1). While for some categories, the informational needs for technical aspects are more accentuated (for investors), for others the discussions need to be heavier oriented to disseverances (for regulatory bodies), while for others the communication strategy needs to be mainly focused on advantages over competing technologies (for medics and patients). A one-size-fits-all approach is thus not optimal. Therefore, strategic communication must be segmented and calibrated to engage each category in the most effective way, ensuring that the message is finely tuned not only from the content point of view, but also in terms of delivery means. In the next we outline some aspects that need to be considered when adjusting the employed communication strategies to several relevant stakeholder categories. These stakeholder groups are discussed separately in the next four subsections because they evaluate emerging technologies through distinct decision frameworks and evidentiary expectations. These differences are summarized in Table 2, which highlights the main communication priorities associated with each stakeholder group.

In addition, in the second part of this Section we discuss a series of key tools that can significantly augment communication workflows. It should be noted that such tools are illustrative rather than exhaustive, and are intended to demonstrate how strategic communication principles can be operationalized in practice.

**Table 2:** Comparative decision frameworks and communication priorities across key stakeholders in the translation of bionanomaterials

Stakeholder	Primary decision logic	Risk tolerance	Evidence expectations	Key communication priorities
Patients and patient communities	Personal health benefit and safety	Low	Clear clinical outcomes and safety data	Transparent explanation of benefits, risks, and realistic expectations
Clinicians and healthcare providers	Clinical utility and integration into practice	Moderate	Clinical trial evidence, comparative performance, usability	Demonstration of therapeutic value, workflow compatibility, and patient outcomes
Regulators	Safety, efficacy, and regulatory compliance	Very low	Robust preclinical data, controlled clinical evidence, reproducibility	Transparent reporting, risk–benefit assessment, and adherence to regulatory standards
Investors and industry partners	Market potential and scalability	Higher	Technology readiness, intellectual property position, development milestones	Value proposition, differentiation from existing solutions, and commercialization pathway

### Patients: Focus on plain-language science and digital storytelling

Patients represent the ultimate end-users of clinical innovations. However, most of the times they are overlooked in science-driven communication. For functional bionanomaterials, it is important to take into account that patients may be concerned by terms such as “nanotechnology” or “synthetic implants” which they may not properly understand; additional examples are provided in Table 3.

**Table 3:** Specialized terms in the field of bionanomaterials that may be difficult for patients to understand

Term	Why It’s Difficult
Functionalized nanoparticles	Unclear what “functionalized” means or what functions are being added.
Targeted drug delivery	The term sounds simple, but how ‘targeting’ actually works at the nano scale can be confusing.
Stimuli-responsive materials	Patients may not understand what kind of stimuli (e.g., pH, temperature, light) are involved or how materials ‘respond’.
Bioconjugation	A highly technical term for linking biomolecules to nanomaterials.
Theranostics	A portmanteau of ‘therapy’ and ‘diagnostics’ that’s unfamiliar and jargon-heavy.
Nano-bio interface	Abstract and unclear what interaction this term refers to (e.g., between cells and nanomaterials).
Quantum dots	Misleading name that is often confused with physics or computers, and lacks context in a medical setting.
Liposomes/micelles/dendrimers	Nanoscale carrier structures with unfamiliar and scientific names.
Controlled release kinetics	“Kinetics” is a technical concept; patients don’t usually consider how drugs are released over time.
Bioavailability enhancement	The concept of increasing how much drug is absorbed may be unfamiliar and abstract.
Biodegradable nanocarriers	May raise concerns if patients don’t understand what these carriers are and how they break down in the body.
Cytotoxicity/immunogenicity	Scientific terms for harmful effects or immune reactions, which can alarm patients if not properly contextualized.
Translational nanomedicine	“Translational” is jargon to the public, often misinterpreted as language-related or vague.

Thus, it is particularly important to provide clear and emotionally intelligent communication for this category. Specifically, patient-centered communication should use plain language to describe how a technology works. Also, it should be made clear in easy-to-understand terms what benefits it offers compared to other existing solutions, and what risks it mitigates. Ideally this should be done through visual aids or explainer animations, to avoid technical jargon, which is not always easy to reproduce textually in common terms. The advantages of animations in science education is widely acknowledged [132–134], and similar strategies can potentially be successfully employed to enhance the awareness of patients on specific terms, and mechanism routes of bionanomaterials.

Digital storytelling, such as patient scenarios or testimonials (either real or simulated), can be very useful to help personalize the innovation and to reduce the level of abstraction [135, 136]. It is also important to consider, that trust in a given product is also enforced when the communication is transparent about the regulatory status, trial involvement, and scientific uncertainties. Such aspects should, however, be discussed in potential digital storytelling workflows in easy to grasp terms, otherwise they can raise questions, instead of providing answers.

The patient-oriented communication can be delivered by different means. For example, social media platforms and disease-specific online communities (e.g., rare disease forums or cancer support groups) can represent viable solutions to raise awareness on novel solutions & developments and to gather feedback [137–139]. While more difficult to implement, co-creation sessions, where patients are invited to share their perspectives during early development phases, can be as well highly important. Not only that they can improve the design of an application under development but can also strengthen the eventual communication campaign.

### Clinicians: Focus on evidence-based narratives and trust anchoring

Clinicians are pragmatists [140, 141]. Their interest in innovation is filtered through clinical relevance, patient outcomes, integration ease, and safety profiles. As such, communication targeting clinicians should foreground evidence and utility. This includes not only peer-reviewed publications in respected medical journals but also curated data summaries, clinical case studies, and visual infographics that convey comparative efficacy.

Strategically, engaging clinicians through continuing medical education (CME) platforms and programs, and via professional societies can serve as trust-building channels. Collaboration with early clinical adopters and key opinion leaders (KOLs) to co-author perspectives or participate in symposia can potentially be highly efficient in creating validation loops. Furthermore, we argue that framing bionanomaterial technologies as tools that enhance, rather than disrupt, existing workflows, particularly in high-burden specialties like oncology or infectious diseases, can be highly important to consider for increasing significantly the clinician receptivity.

Communication strategies targeting clinicians should consider key aspects that make them reluctant in adopting novel/disrupting (nano)technologies, Table 4. This reluctance usually stems from a combination of factors such as: limited clinical evidence, unfamiliarity with complex scientific concepts, and concerns over safety and long-term effects. Regulatory ambiguity and the lack of standardized guidelines further contribute to uncertainty. Additionally, integration challenges into existing clinical workflows, the absence of reimbursement pathways, and limited patient awareness or demand, make adoption less attractive. Ultimately, risk aversion and a preference for established, well-understood treatments play a major role in slowing clinical uptake [24]. 

**Table 4:** Typical reasons for clinician reluctance to adopt disruptive bionanotechnologies

Reason	Explanation
Limited clinical evidence	Lack of large-scale, long-term human data proving safety and efficacy.
Scientific complexity	Concepts like stimuli-responsive carriers or functionalized nanoparticles are poorly understood by many clinicians.
Regulatory ambiguity	Unclear classification between drug, device, or biologic leads to confusion and hesitancy.
Integration challenges	New tools or workflows may be required, disrupting clinical routines.
Safety concerns	Unknown long-term effects, biodistribution, or immunogenicity raise caution.
Lack of reimbursement	If not reimbursed, new technologies are less likely to be used in practice.
Limited patient awareness	Patients may not ask for these innovations or may mistrust them due to poor understanding.
Resistance to change	Clinicians often prefer established, proven treatments over untested innovations.

### Regulators: Focus on clarity, consistency, and collaborative engagement

Regulatory bodies require clarity, consistency, and foresight in communication. Given that many bionanomaterials sit at the intersection of distinct categories, such as devices, biologics, and pharmaceuticals, early and iterative engagement is essential. Equally important is the use of multimodal presentation formats, combining text, visuals, and data-driven infographics, which support deeper cognitive encoding, reduce abstraction barriers, and engage both verbal and non-verbal processing systems [142–144]. Such approaches make complex mechanisms of action, risk–benefit profiles, and safety data more accessible and interpretable to regulatory reviewers. By integrating visual and narrative elements within technical documentation, developers can significantly enhance the precision, transparency, and persuasiveness of their regulatory interactions.

Effective communication strategies here include the development of clear regulatory briefing documents, structured risk-benefit analyses, and alignment with existing guidance documents (e.g., EMA or FDA frameworks), Table 5. Visual schematics that explain mechanisms of action, degradation pathways, and safety margins can help regulators assess complex platforms more effectively.

**Table 5:** Key aspects that need to be addressed in the discourse of bionanomaterials developers to regulatory bodies

Aspect	Measures
Clear mechanism of action	Precisely describe how the bionanomaterial interacts with biological systems.
Safety and toxicological profile	Include detailed data on cytotoxicity, immunogenicity, biodistribution, and long-term effects.
Manufacturing and quality control	Demonstrate reproducibility, scalability, and compliance with GMP standards.
Characterization and standardization	Provide physicochemical properties (size, charge, composition) and ensure batch consistency.
Preclinical efficacy	Robust in vitro and in vivo data showing biological effect in disease models.
Regulatory classification justification	Clearly define whether the product is a drug, device, biologic, or combination and justify classification.
Clinical translation strategy	Present a pathway to clinical trials including trial design, endpoints, and patient safety considerations.
Risk-benefit analysis	Quantify potential risks versus therapeutic benefits based on available data.
Regulatory precedents	Reference comparable approved nanomedicines or regulatory guidelines, if applicable.
Post-market surveillance plan	Outline strategies for monitoring safety and effectiveness after approval.

In some cases, precompetitive collaboration among developers to establish standardized terminology, testing protocols, or reference datasets can ease regulatory scrutiny. In such joint efforts, addressing emerging, still largely uncharted, aspects such as the insurability of nanomaterials [145], will also be highly important. Such collective efforts not only de-risk individual pipelines but also elevate the legitimacy of the entire field. Participating in or initiating regulatory science consortia may also serve as a communication channel and influence policy evolution, so such initiatives also need to be considered as key tools in the efficient and successful communication with regulatory bodies worldwide.

### Investors: Focus on translational readiness and value proposition framing

Investors prioritize scalability, differentiation, and return on investment [146–148]. While fascinated by innovation, they are typically more attuned to de-risked development pipelines than to raw scientific novelty. Therefore, communication must emphasize aspects like technology readiness levels (TRLs), preclinical validation, IP status, and projected time-to-market, Table 6.

**Table 6:** Key aspects that need to be addressed in the discourse of bionanomaterials developers to investors

Aspect	Measures
Clear value proposition	Describe the unique advantage the bionanomaterial offers over existing solutions.
Market need and size	Demonstrate a significant unmet clinical need and a sizable, accessible market.
Competitive differentiation	Explain how the technology stands out from existing or emerging competitors.
IP position and freedom to operate	Detail patents filed, granted, and any IP barriers to commercialization.
Development timeline and milestones	Provide a roadmap with clear technical, regulatory, and commercial milestones.
Team expertise	Highlight the strength and experience of the scientific, regulatory, and business teams.
Clinical and regulatory pathway	Outline the strategy for regulatory approval and the path to clinical validation.
Manufacturing and scalability	Explain feasibility of cost-effective production at scale.
Exit strategy and ROI potential	Include potential exit routes such as acquisition, licensing, or IPO, and projected returns.
Risk mitigation strategy	Address foreseeable scientific, regulatory, or market risks and how they are managed.

In practice, translating complex scientific advances into investor-oriented narratives often involves collaboration with technology transfer offices, business development teams, incubators, or other commercialization intermediaries [50, 149], which help bridge the gap between scientific and economic language. These actors frequently assist in preparing materials such as investor decks, market analyses, and narrative-driven business cases that contextualize innovations within clinical and commercial landscapes. Such materials should contextualize the innovation within clinical and commercial landscapes placing clear emphasis on objective measures: (i) what problem does the technology solve, (ii) what is the size of the addressable market, and (iii) how the technology outperforms current standards [150].

Additionally, developing modular communication assets (e.g., one-pagers, explainer videos, and ROI visualizations) that can be adapted for venture capitalists, government funders, or strategic partners will be key. Furthermore, technology developed should place important focus on participation in pitch competitions, biotech incubators, and demo days, not only to provide exposure to the proposed tools and solutions but also to gather informed feedback, that will be highly useful for refining communication strategies and align with market expectations [50, 150–152].

### Digital platforms and content strategies for science communication

In the digital era, the traditional boundaries between scientific research and public engagement are dissolving [153–156]. Researchers and innovators developing functional bionanomaterials now have unprecedented access to platforms and tools that can enhance visibility, foster trust, and accelerate stakeholder engagement [157–159]. However, to leverage these tools effectively, communication efforts must be intentional, data-driven, and aligned with the strategic goals of translational success. Recent experience during the COVID-19 pandemic highlighted the transformative power of digital communication in shaping public perception and trust toward novel biomedical technologies [160]. Twitter threads and YouTube explainers from trusted scientists played a pivotal role in countering misinformation and fostering confidence in emerging technologies such as mRNA vaccines [161]. In Spain, for example, daily press conferences by Fernando Simón, Director of the National Center for Health Alerts and Emergencies, demonstrated how consistent, transparent, and didactic messaging can sustain public attention and trust, even when not free from controversy, underscoring the societal value of visible, credible scientific voices [162]. These lessons are directly relevant to the bionanomaterials community, where public understanding and stakeholder confidence will similarly determine the speed and scale of clinical adoption.

**Fig. 4:**
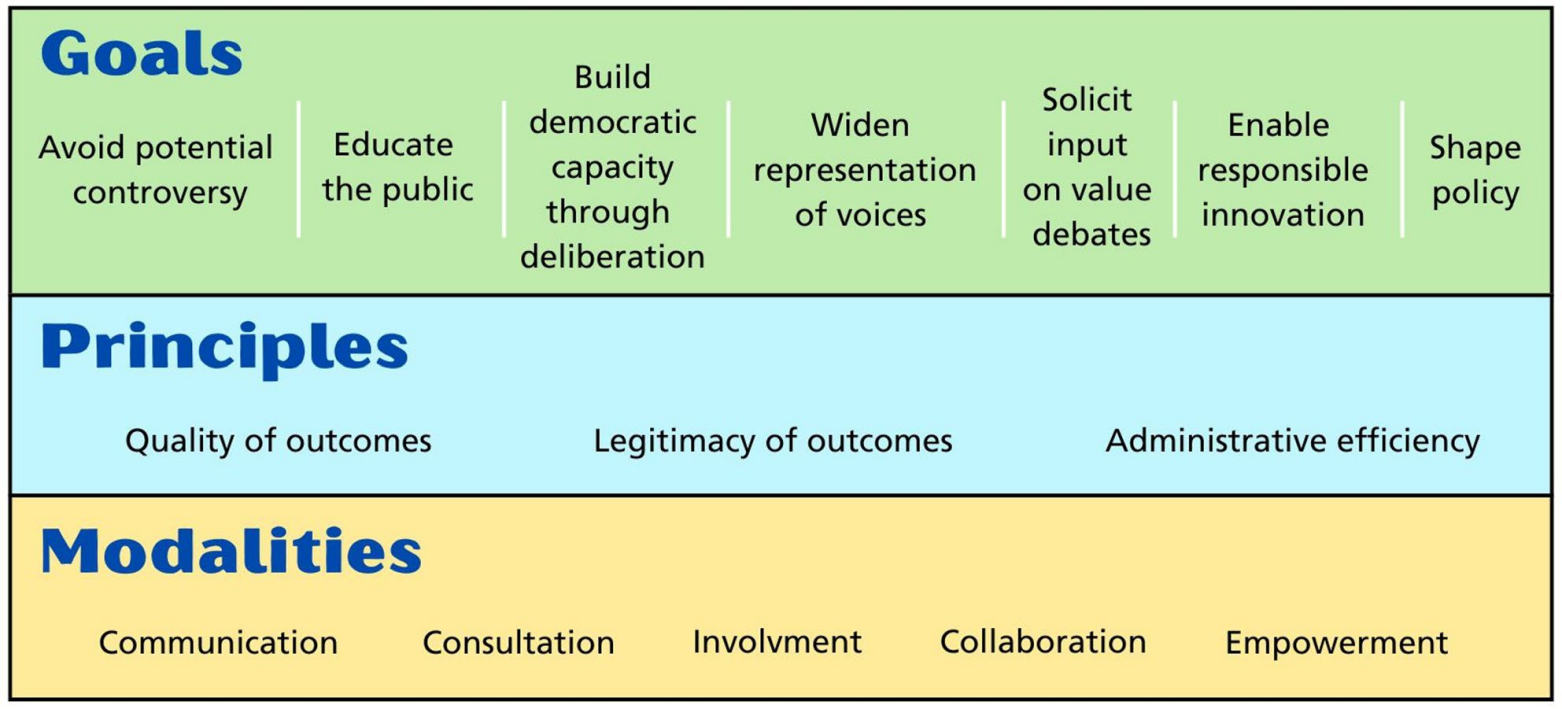
Goals, principles, and modalities of effective public engagement (reproduced from [155], with permission from PNAS)

Concerning the goals of public engagement efforts, in a recent study [155] assessing the likely successes of various modalities for enhancing public engagement of the Nobel prize awarded CRISPR gene editing nanotechnology [163], the authors propose a framework that outlines seven goals of public engagement, arranged according to the degree to which various publics can meaningfully influence policy or the scientific endeavour, Fig. 4. We find that these goals align perfectly with those that need to be addressed in the context of designing and implementing public engagement modalities tuned to the specifics of bionanomaterials and other nanotechnologies for medicine.

Communication strategies must also account for the growing prevalence of misinformation [164–167] and oversimplified narratives circulating across digital platforms. Scientific content often competes with highly amplified non-expert voices, sometimes with a financially motivated discourse [165], that may distort or misrepresent emerging technologies. Addressing this challenge requires proactive engagement from trusted and visible scientific institutions and experts, along with the development of clear, accessible communication formats capable of effectively competing within modern digital media environments. Last, it is important to note that research on medical misinformation is rapidly expanding, and improving our understanding on how such narratives emerge and propagate, sometimes by carefully crafted schemes [168], will be essential for developing effective counterstrategies in the years to come.

### Social media and science branding

Platforms like X (formerly Twitter), LinkedIn, Bluesky, YouTube, and even TikTok or Facebook, have become powerful dissemination channels for scientific content, offering real-time engagement with clinicians, policymakers, industry leaders, and patients [157, 159, 169–173]. Strategic use of these platforms, through regular updates, visual abstracts, and behind-the-scenes lab content, can humanize science and make bionanomaterial research more approachable and memorable. Furthermore, in a Letter published in Science in 2017, the authors emphasize on the fact that scientists need social media influencers, who can play an important role in determining citizens to embrace more rational and scientific-based attitudes [174]. A more recent study shows the role of science influencers in influencing supportive and skeptical communities on politicized science subjects (e.g. climate change, COVID-19 pandemic, etc.) [175]. In light of these emerging trends, a key challenge in designing social media campaigns augmented by influencers, is identifying the appropriate individuals; an analysis in this regard has been presented by Bethu et al. [176]. Also important to highlight is the study of Buitrago et al. [177] who show the added value of science dissemination on YouTube by influencers, instead of institutional channels. The body of literature on the role of social media influencers on promoting science is consistently expanding, and such trends need to be considered also for forthcoming social media campaigns of bionanomaterials developers. In this context, it is important to recognize that the rise of online “medical influencers” illustrates both opportunities and risks: while credible scientific voices can reach large audiences, engaging but non-evidence-based narratives may also spread rapidly within algorithm-driven information ecosystems [178]. In such environments, credible scientific voices risk being overshadowed by more visible but less reliable sources. Consequently, effective communication strategies must not only focus on accuracy but also on reach and engagement, ensuring that trustworthy information can effectively circulate within broader public discourse. A growing body of research focuses on how algorithms contribute to misinformation spread [179, 180]. Achieving a correct understanding of these mechanisms, will be instrumental in developing strategies that promote the dissemination of scientifically valid information over misleading or inaccurate narratives.

**Fig. 5:**
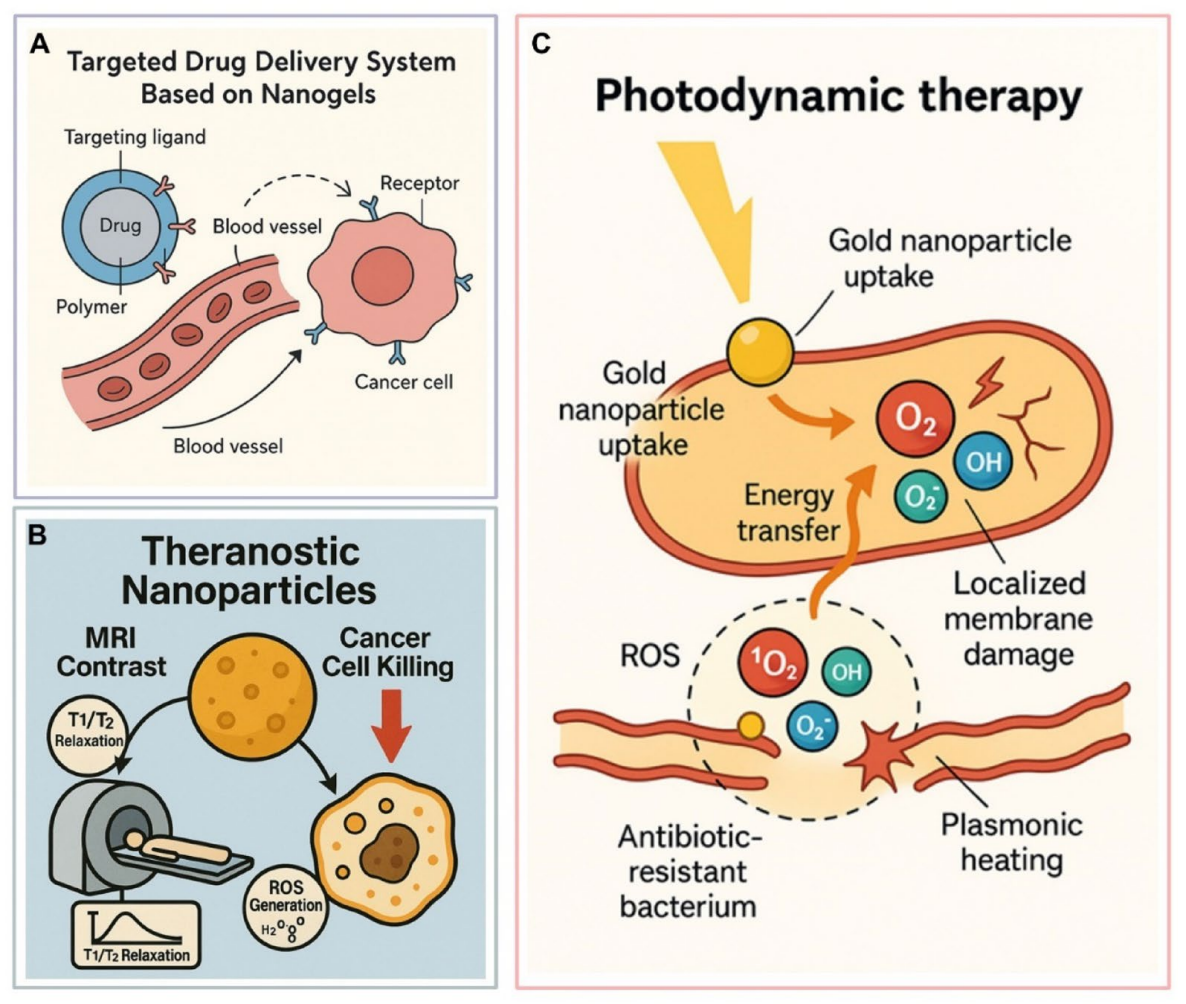
Conceptual drawings reflecting various biomedical applications of bionanomaterials generated on the fly by ChatGPT using the syntax: (**A**) “*Draw concept figure for: targeted drug delivery system based on nanogels*”. (**B**) “*Draw concept figure for theranostic nanoparticles for MRI contrast and cancer cell killing”* followed by revision request to *“add labels*,* arrows*,* and mechanistic insets (e.g.*,* T1/T2 relaxation*,* ROS generation)*”; (**C**) “*Draw a conceptual figure for photodynamic therapy with gold nanoparticles of antibiotic resistant bacteria*,* illustrating the uptake of the gold nanoparticles and the effects of the light excitation”* followed by revision request to represent it as a “*mechanistic version showing ROS generation (¹O₂*,* •OH*,* O₂•–)*,* localized membrane damage*,* plasmonic heating from AuNP excitation*,* energy transfer diagram (light → AuNP → ROS)*”

Hashtag campaigns, such as #NanoInMedicine or #SmartMaterials, can help position technologies within larger scientific movements. Researchers can also co-brand their work using consistent visuals, icons, or terminology that reinforce recognition. For example, a targeted drug delivery system based on nanogels could be framed not just as “novel” but as a “next-gen precision payload for immune modulation,” offering a clear narrative hook. To present a graphical representation, that can be easy to grasp, a simple way is to employ emerging Large-Language Models such as ChatGPT, DeepSeek, etc. With the help of these a conceptual drawing figure for this topic can be generated in minutes, same as for other medical applications of bionanomaterials (Fig. 5). Such tools, have also been identified as important tools for science education, including in nanotechnology [181, 182].

While AI-assisted graphical generation tools can significantly accelerate the production of illustrative scientific content in a highly facile manner, it should be noted that at their current maturity stage such tools are also prone to introduce visual artifacts or annotation inconsistencies. For example, Fig. 5C displays typical limitations of current generative approaches, including duplicated labels (“gold nanoparticle uptake”), minor annotation errors (e.g. the missing number in the O₂ label), and inconsistencies in the color representation of chemical species. These limitations are particularly relevant when depicting biochemical mechanisms or nanomaterial–biological interactions, where small graphical inaccuracies may lead to conceptual misunderstandings. Therefore, although current AI tools can support rapid visual prototyping, careful expert review and manual refinement remain essential to ensure scientific accuracy and clarity in final graphical outputs. In a preprint published in 2025 by Chang et al. [183], the authors introduced *SridBench*, a benchmark designed to evaluate multimodal models in scientific figure generation across multiple disciplines. Their findings indicate that, with the exception of GPT-4o-image, most evaluated models lack robust capabilities for accurate scientific representation. Even for GPT-4o-image, limitations were observed to persist, including incomplete textual information, missing visual elements, and occasional hallucinations or common-sense errors. Besides technical issues with LLM generated data, the recent Opinion paper of Marushchenko et al. [184], raises also important considerations regarding authorship, intellectual property, and copyright when using AI-assisted tools for scientific image generation, emphasizing the need for careful validation and responsible use of such outputs. The authors also provide a relevant summary of selected journal and publisher policies on AI-generated images.

### Explainer videos and infographics

Complex ideas like nano-bio interfaces, stimuli-responsive behaviour, or hybrid nanostructures often defy quick explanation. Visual content, particularly short, animated explainer videos or dynamic infographics, can bridge this comprehension gap. These tools are especially effective for conveying mechanisms of action, comparative advantages, and patient impact. Same as proposed under the previous point, besides more elaborate approaches that rely on collaborations with science illustrators or communication agencies familiar with biomedical contexts, generative AI can represent a quick and efficient tool in providing such materials, based on simple instructions (Fig. 5). While ChatGPT currently does not straightforward support creating videos, it can generate scripts, generate individual animation frames or mock-ups to aid an experienced artist. High-quality visual content can potentially be embedded in grant proposals, investor decks, or shared on institutional websites and media platforms, with the role of visual representation for explaining and promoting science being well acknowledged to date [133, 185, 186].

### Preprint platforms and lay summaries

Publishing on preprint servers such as *bioRxiv*, *chemRxiv* or *medRxiv* provides early visibility and enables timely feedback from a broader audience [187–189]. However, generally preprints consist of the version submitted for evaluation to a journal, which hinders the impact of early dissemination. Technical jargon and cautious framing of results, highly specific to manuscripts presenting work on bionanomaterials and nanotechnology topics, can limit accessibility and hinder broader translational impact.

In their recent work Goldstein and Krukowski [190], argue that when accompanied by plain-language summaries or lay abstracts, scientific publications become more accessible to non-specialist stakeholders. This idea was also previously advocated by Kuehne and Olden [191]. Motivated by this, some journals now publish lay summaries next to the scientific article. We argue that these lay summaries need to be prepared from the very early stage of preprint publishing, to promote impact of the novel technology. Some useful hints provided by Goldstein and Krukowski [190] such as the use of simple sentences, positive framing, or consulting with external stake-holders prior publication, are indeed highly useful to consider, alongside other guidelines [192–195].

This layered approach, combining rigorous data with digestible messaging can significantly stimulate dialogue and interest even before peer-reviewed publications are ready, which can take in the range of months to years, considering the typical manuscript handling times for editorial screening, and for peer-review and editorial decision [196, 197]. We argue that key attention also needs to be placed on “graphical takeaways”, that many journals invite as “Graphical Abstracts”, and on “Twitter summaries,” both representing valuable tools for researchers to deliver compelling microcontent that drives broader engagement.

### Search engine optimization (SEO) for research visibility

Optimizing digital content for search engines ensures that key audiences can find relevant information when they need it. While SEO is a very common practice for generic websites, it has only been recently employed for enhancing accessibility to scientific outputs, such as journal articles [198, 199]. Besides employing such approaches for promoting access to scientific publications, they also need to be thoroughly considered for complementary purposes. For example, a microsite for a research grant presenting the development of a responsive nanocarrier should include searchable keywords like “targeted drug delivery for inflammatory disease” or “biodegradable nanogels for wound healing.” Strategic tagging, backlinking to publications, and use of descriptive meta-content all contribute to improving discoverability and need to be carefully employed. Additionally, research groups can also benefit from publishing whitepapers, FAQs, and multimedia blogs tailored for specific audiences, positioning themselves not only as innovators but also as thought leaders.

### Collaborations with medical influencers and key opinion leaders

While in previous subsections the concept of medical influencers was discussed in the context of the dynamics of information dissemination across digital platforms, this section highlights longer-term communication approaches that foster sustained engagement with scientific communities and stakeholders. Key opinion leaders (KOLs) and digital medical influencers are increasingly shaping conversations around emerging technologies, as discussed also in the previous. The instrumental role of KOLs for the adoption of new medicine, and new therapeutic approaches is well acknowledged, as outlined in a recent perspective published in Nature Reviews Rheumatology [200]. Strategic collaborations, such as co-hosting webinars, participating in podcast interviews, or contributing to joint blog posts, can help amplify bionanomaterials to targeted professional or patient communities [201–203]. These partnerships should be framed as knowledge-sharing rather than promotion, and care must be taken to preserve scientific integrity. We argue that when executed thoughtfully, such collaborations have significant potential to extend the reach of novel bionanomaterials/technologies and support/augment their reputational capital.

It is also important to acknowledge however that the increasing prevalence of monetized partnerships and sponsored content across digital platforms introduces additional challenges for science communication, including in the frame of communication strategies embedding influencers and KOLs. In some cases, undisclosed financial relationships, promotional campaigns, or even the dependence of the research agenda of a scientist on industry sponsorship [204], may amplify products or technologies that lack robust scientific validation. Such practices risk misleading audiences and may ultimately erode trust in legitimate innovations by blurring the boundary between evidence-based communication and commercial promotion. We argue thus that ensuring transparency in sponsorship disclosure and maintaining strong standards of scientific integrity are therefore essential for preserving credibility in the communication of emerging biomedical technologies. The importance of transparency and disclosure in medical communication has long been recognized, as discussed in a seminal commentary by Davidoff et al. published in *The Lancet* [205], underscoring the enduring relevance of addressing sponsorship and conflicts of interest in scientific discourse.

## Strategic communication pathways for the translation of functional bionanomaterials

**Fig. 6:**
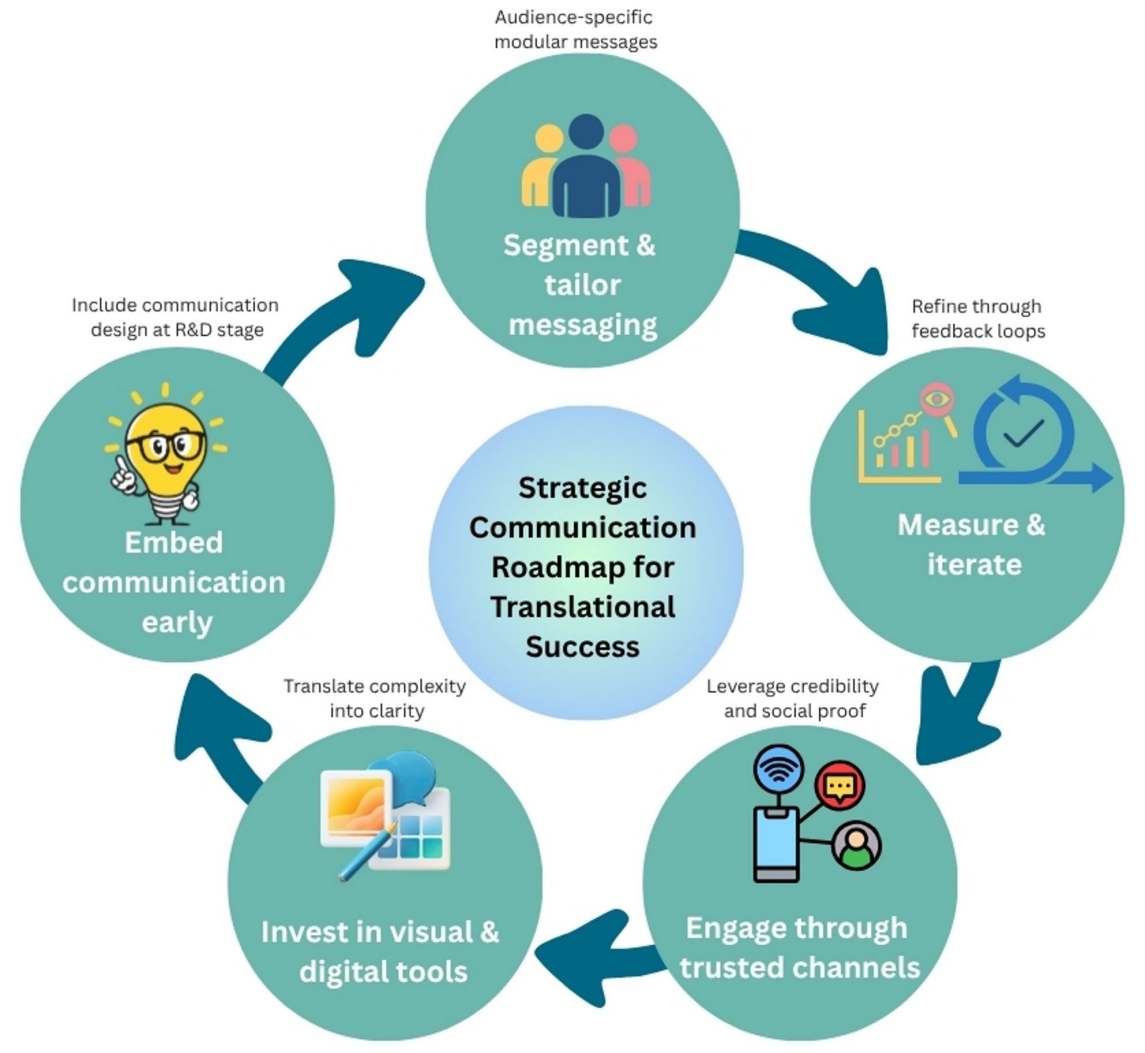
Strategic communication roadmap for translational adoption of functional bionanomaterials

In this Perspective, we highlighted so far how strategic communication, when grounded in authenticity, clarity, and stakeholder-centric design, can serve as a powerful tool to bridge the persistent gap between innovation and adoption of smart, advanced, bionanomaterials and of innovative nanomedicine approaches. By integrating core principles of positioning, narrative construction, visual storytelling, and audience segmentation, researchers and innovators can proactively shape the perception, relevance, and acceptability of bionanomaterials within the clinical ecosystem. To operationalize this approach, we propose the following communication roadmap, outlined also in Fig. 6:

1. **Embed communication early**: Integrate stakeholder mapping and messaging strategies at the R&D planning stage, not post hoc. Consider communication a design parameter, not an afterthought. This reduces later framing conflicts and reactance, phenomena well-documented in communication psychology [206].
2. **Segment and tailor messaging**: Develop modular, audience-specific, communication assets that speak directly to the priorities of clinicians, patients, regulators, and investors, and thus increase message efficacy through relevance.
3. **Invest in visual and digital tools**: Utilize infographics, explainer videos, and plain-language summaries to translate complexity into clarity and increase engagement across platforms.
4. **Engage through trusted channels**: Partner with professional societies, KOLs, and patient communities to deliver messages through credible, established networks, thereby activating social proof mechanisms, where endorsement by respected peers reinforces trust and adoption among wider audiences.
5. **Measure and iterate**: Treat communication as a dynamic process and use analytics, feedback loops, and stakeholder input to refine strategy and improve resonance.

Last, we discuss ways to measure the impact of strategic communication in bionanomaterials translation. While communication outcomes are often perceived as qualitative or subjective, a growing body of translational and implementation research [207–209] demonstrates that structured indicators can be used to assess communication effectiveness across stakeholder groups.

At the preclinical and early translational stages, impact can be evaluated through indicators of stakeholder awareness and comprehension. These include changes in clinician or regulator understanding of a technology’s mechanism of action, safety profile, and intended clinical use, assessed through surveys, structured interviews, or feedback during advisory meetings. Metrics such as clarity of perceived value proposition and reduction in misinterpretation of technical terminology provide early signals of communication alignment. At later translational stages, trust-related indicators become increasingly important. These may include regulator feedback cycles, frequency of clarification requests during regulatory interactions, willingness of clinicians to engage in first-in-human or early-phase trials, and the presence of key opinion leader endorsement. In parallel, patient-facing communication can be evaluated through measures of informed consent quality, patient-reported understanding, and acceptance of trial participation. Ultimately, downstream translational outcomes offer additional quantitative proxies for communication effectiveness. These include progression through technology readiness levels, time-to-clinical trial initiation, adoption rates in pilot clinical settings, and post-approval uptake relative to comparable technologies. Importantly, these indicators should not be interpreted in isolation but analyzed in conjunction with technical and regulatory factors. Table 7 summarizes representative indicators that may be used to assess the effectiveness of strategic communication across key stakeholder groups involved in the translation of functional bionanomaterials.

**Table 7:** Conceptual dimensions and indicative metrics for evaluating the effectiveness of communication strategies in the translational pathway of bionanomaterials

Stakeholder group	Communication objectives	Representative assessment indicators	Translational stage(s)
**Clinicians**	Clarify clinical value, safety, and workflow integration	• Changes in perceived clinical relevance • Willingness to participate in early-phase trials • Adoption in pilot or compassionate-use settings • Feedback during clinical advisory boards	Preclinical → early clinical
**Patients/Public**	Improve understanding, trust, and informed consent	• Patient-reported understanding of risks and benefits • Enrollment and retention in clinical trials • Quality of informed consent processes • Public trust indicators (surveys, engagement metrics)	Clinical trials
**Regulatory authorities**	Reduce uncertainty and align on safety and benefit–risk profiles	• Frequency and nature of clarification requests • Duration of regulatory review cycles • Consistency of regulatory feedback across interactions • Acceptance of proposed endpoints	Preclinical → regulatory submission
**Investors/Funding bodies**	Communicate feasibility, differentiation, and translational readiness	• Investment interest or funding success • Alignment on development milestones • Continuity of financial support across stages • Perceived translational credibility	Early translation
**Industry partners**	Enable technology transfer and scalability	• Initiation of licensing or partnership discussions • Progression toward scale-up agreements • Integration into existing development pipelines	Late preclinical → translation
**Multistakeholder ecosystem**	Foster alignment and coordinated decision-making	• Cross-stakeholder consensus on value proposition • Reduction of conflicting expectations • Acceleration of progression through translational milestones	Across stages

We argue that embedding such evaluation metrics into translational workflows enables iterative refinement of communication strategies, transforming communication from an ad hoc activity into a designable and accountable component of nanobiotechnology development. This approach aligns with emerging views of translation as a socio-technical process, in which scientific performance and stakeholder understanding co-evolve.

It should be noted however that the metrics summarized in Table 5 represent conceptual dimensions for evaluating communication effectiveness rather than a single standardized measurement framework. In practice, these dimensions can be assessed using a variety of methodological approaches commonly employed in science communication and implementation research. From early foundational work, such as the landmark study of D. Edge in 1979 [210] which explored methods to quantify scientific communication effectiveness, to more recent frameworks reviewed by B. Fischhoff [211], the range of available evaluation approaches has significantly evolved. Recent interdisciplinary studies [212] further highlight the role of cognitive and behavioral factors, including information processing mechanisms, trust formation, and cognitive biases, in shaping how scientific messages are received and interpreted. Current quantitative measures include: (i) stakeholder surveys measuring trust, perceived risk, and technology acceptance; (ii) engagement analytics derived from digital communication platforms; (ii) qualitative feedback from stakeholder consultations; and (iv) indicators related to technology adoption or stakeholder alignment. Together, these approaches can provide complementary evidence for assessing the effectiveness of communication strategies across different stages of the translational pathway. Similar multidimensional evaluation approaches are commonly used in implementation science to assess stakeholder engagement and adoption processes [213, 214].

## Limitations and Responsible Use of Communication Strategies

While this work emphasizes the importance of strategic communication in supporting the translational trajectory of bionanomaterials, it is important to recognize that communication does not operate independently of other determinants of innovation adoption. Technical maturity, rigorous experimental validation, regulatory facilitation, and market dynamics remain fundamental drivers of lab-to-clinic science translation. Rather, communication acts as a cross-cutting enabler that interacts with these factors by shaping how stakeholders interpret technological readiness, risk–benefit profiles, and clinical value propositions, as discussed under the previous sections.

Through mechanisms such as trust formation, risk framing, and stakeholder engagement, communication strategies can influence how emerging technologies are evaluated by regulators, clinicians, investors, and the public. However, these strategies must be implemented responsibly. Communication practices that prioritize promotional narratives over evidence-based messaging may contribute to unrealistic expectations regarding technological readiness and potentially reinforce hype cycles in rapidly evolving scientific fields [127–129].

In addition, the increasing role of digital communication ecosystems introduces further ethical considerations, including the influence of monetized partnerships, undisclosed sponsorships, and the rapid amplification of misleading information [168, 178]. This is further accentuated by aspects such as those presented by Vosoughi et al. [215], who discuss in their study published in *Science* that false information spreads significantly faster, deeper, and more widely on social networks (their study addressing Twitter) than fact-based information across all categories. The authors concluded that this occurs not because the information is false per se, but because falsehoods tend to appear more novel, unusual, or surprising than truths [215]. This has been further confirmed by later studies [127]. These dynamics are indeed prone to create tensions between dissemination goals and scientific rigor, particularly when complex findings are simplified for broader audiences. Maintaining transparency, clearly communicating technological limitations, and prioritizing scientific integrity are therefore essential to ensure that communication strategies support, rather than undermine, the responsible development and adoption of novel bionanomaterials [114, 115].

Finally, it should be acknowledged that this work represents a perspective-based synthesis of the literature rather than a systematic review. As such, the selection and interpretation of examples may not be exhaustive and reflect the authors’ emphasis on illustrative case studies and conceptual framing. Future work could benefit from more systematic and quantitative analyses to further validate and refine the proposed communication strategies.

## Conclusion

Functional bionanomaterials hold substantial transformative potential across a wide range of biomedical and clinical applications, and novel design and synthesis routes, accompanied by novel high impact applications in nanomedicine emerge on a daily base. However, as the field matures from fundamental discovery toward translational implementation, it is increasingly evident that clinical success depends not only on physico-chemical performance, safety, and regulatory compliance, but also on how these technologies are communicated, interpreted, and trusted by diverse stakeholder groups.

In this Perspective, we have argued that strategic communication should be recognized as a core, designable component of the translational pipeline for functional bionanomaterials, rather than a downstream dissemination activity, as currently handled by many research groups worldwide. By synthesizing insights from nanomedicine translation, behavioral science, and innovation studies, we have outlined a structured framework and practical roadmap that position communication alongside scientific development and regulatory planning. Importantly, we have also highlighted measurable indicators that enable communication effectiveness to be evaluated and iteratively refined across translational stages.

Viewing translation as a socio-technical process underscores the need for bidirectional engagement between developers, clinicians, patients, regulators, and investors. Such dialogue not only facilitates trust and adoption but can also inform research priorities, clinical relevance, and implementation strategies. Achieving this level of integration requires institutional support, dedicated expertise, and sustained resources, rather than reliance on individual researchers alone.

Ultimately, the successful clinical integration of functional bionanomaterials will depend on aligning technical innovation with stakeholder understanding and confidence. By embedding strategic communication into translational workflows, the nanobiotechnology community can help ensure that scientifically robust bionanomaterials are not only developed, but are also effectively positioned to reach, and benefit, patients in clinical practice.

## Data Availability

No datasets were generated or analysed during the current study.
